# Budding and explosive membrane vesicle production by hypervesiculating *Escherichia coli* strain Δ*rodZ*

**DOI:** 10.3389/fmicb.2024.1400434

**Published:** 2024-06-20

**Authors:** Yoshihiro Ojima, Kaho Toda, Tomomi Sawabe, Yuki Kumazoe, Yuhei O. Tahara, Makoto Miyata, Masayuki Azuma

**Affiliations:** ^1^Department of Chemistry and Bioengineering, Graduate School of Engineering, Osaka Metropolitan University, Osaka, Japan; ^2^Graduate School of Science, Osaka Metropolitan University, Osaka, Japan

**Keywords:** *Escherichia coli*, outer membrane vesicle, Δ*rodZ*, CRISPRi, quick-freeze deep etch replica electron microscopy, budding

## Abstract

*Escherichia coli* produces extracellular vesicles called outer membrane vesicles. In this study, we investigated the mechanism underlying the hypervesiculation of deletion mutant Δ*rodZ* of *E. coli*. RodZ forms supramolecular complexes with actin protein MreB and peptidoglycan (PG) synthase, and plays an important role in determining the cell shape. Because *mreB* is an essential gene, an expression-repressed strain (*mreB*^R3^) was constructed using CRISPRi, in which the expression of *mreB* decreased to 20% of that in the wild-type (WT) strain. In shaken-flask culture, the Δ*rodZ* strain produced >50 times more vesicles than the WT strain. The *mreB*-repressed strain *mreB*^R3^ showed eightfold higher vesicle production than the WT. Δ*rodZ* and *mreB*^R3^ cells were observed using quick-freeze replica electron microscopy. As reported in previous studies, Δ*rodZ* cells were spherical (WT cells are rod-shaped). Some Δ*rodZ* cells (around 7% in total) had aberrant surface structures, such as budding vesicles and dented surfaces, or curved patterns on the surface. Holes in the PG layer and an increased cell volume were observed for Δ*rodZ* and *mreB*^R3^ cells compared with the WT. In conditions of osmotic support using sucrose, the OD_660_ value of the Δ*rodZ* strain increased significantly, and vesicle production decreased drastically, compared with those in the absence of sucrose. This study first clarified that vesicle production by the *E. coli* Δ*rodZ* strain is promoted by surface budding and a burst of cells that became osmotically sensitive because of their incomplete PG structure.

## Introduction

Outer membrane vesicles (OMVs) are nanosized, spherical, bilayered membranous structures with a diameter of 20–250 nm. OMVs are discharged from the surface of Gram-negative bacteria, including *E. coli* (Schwechheimer and Kuehn, 2015; Toyofuku et al., 2019). OMVs contain outer membrane proteins, lipids, periplasmic proteins, lipopolysaccharides, RNA, and DNA.

Vesicle formation is promoted by a disturbance in cell growth, turnover in cell wall components, or exposure to antibiotics (Schwechheimer and Kuehn, 2015; Toyofuku et al., 2019). In addition, previous genome-wide assessment of OMV production by *E. coli* demonstrated that the deletion of genes relating to the envelope structure and phospholipid accumulation in the membrane drastically increased vesicle formation (Kulp et al., 2015). Among the genes identified in that study, deletion of *rodZ* (*yfgA*) resulted in the highest OMV production. However, its hypervesiculation mechanism remains unclear. RodZ is part of the Rod complex, which synthesizes the PG for elongation in *E. coli* cells (Egan et al., 2020). In particular, RodZ is required for proper assembly of the MreB actin cytoskeleton and cell shape (Bendezú et al., 2009). RodZ is not essential for viability, but its loss leads to misassembly of MreB into a non-spiral structure and a consequent loss of cell shape (Shiomi et al., 2008; Bendezú et al., 2009). Cells lacking *rodZ* are spherical (compared with rod-shaped WT cells) and grow slower than WT cells. MreB is an essential actin in the cells, and accumulates just beneath the cytoplasmic membrane in a spiral/banded-like pattern along the long axis of the cell (Bendezú et al., 2009). Clear evidence for an important role of MreB in cell shape maintenance came from the isolation of a spherical *E. coli* mutant (Bendezú et al., 2009). More recent research reported that CRISPR interference (CRISPRi)-mediated repression of *mreB* expression also induced morphological change of cells to spherical (Elhadi et al., 2016). However, although many studies have reported on the relationship between *rodZ* or *mreB* and cell morphology, to our knowledge, no studies have yet focused on their relationship with OMV production.

Meanwhile, we have studied the mechanism of enhanced OMV production in *E. coli* knockout mutant strains in envelope structure (Ojima et al., 2021; Sawabe et al., 2024). In our previous study, cells of *E. coli nlpI* and *mlaE* mutant strains were observed by quick-freeze deep-etch electron microscopy (QFDE-EM) to clarify the mechanisms of enhanced OMV production (Ojima et al., 2021). QFDE-EM revealed that plasmolysis, shrinking of the protoplasm away from the cell wall, occurred at the tip of the long axis in cells of these mutant strains. Plasmolysis was observed more frequently in double-gene deletion mutant Δ*mlaE*Δ*nlpI* cells, and OMVs formed from this tip (Ojima et al., 2021), suggesting that plasmolysis is a key mechanism for enhanced OMV production. Thus, QFDE-EM is a powerful tool for investigating the hypervesiculation mechanism in *E. coli*.

While the *rodZ* deficient strain leads to a loss of cell shape due to the incomplete PG structure, deletion of *nlpI* decreases lipoprotein–peptidoglycan (Lpp–PG) crosslinks: the number of Lpp–PG crosslinks is approximately 40% lower in *nlpI* mutant than in the WT strain (Mcbroom and Kuehn, 2007; Schwechheimer et al., 2015). Deletion of *mlaE* induced phospholipid accumulation in the outer leaflet of the outer membrane (Roier et al., 2016). Therefore, even though these genes are all involved in envelope structure, it is expected that hypervesiculating mechanisms of Δ*rodZ* mutant are different from Δ*nlpI* and Δ*mlaE* mutants.

In this study, we investigated the mechanism underlying the hypervesiculation of deletion mutant Δ*rodZ* and *mreB*-repressed strains of *E. coli*. The surface or cross-section of these cells was observed using QFDE-EM. We also examined the amount and sizes of the cells, and vesicles produced by these strains. Finally, we discuss the possible mechanism of enhanced vesicle production by the Δ*rodZ* mutant.

## Materials and methods

### Bacterial strains and plasmids

The strains and plasmids used in this study were listed (Table 1). The WT *E. coli* K-12 strain BW25113 and *rodZ*-deficient mutant were obtained from the National BioResource Project (National Institute of Genetics [NIG], Mishima, Japan) (Baba et al., 2006). Plasmid pgRNA-bacteria (Addgene ID:44249) was used as a platform vector for the expression of various gRNAs for *mreB* (Qi et al., 2013). The target site complementary sequences of gRNAs in *mreB* were designed according to a previous study (Fang et al., 2021) (Supplementary Figure S1). Custom-designed gRNAs were inserted into pgRNA-bacteria by inverse PCR, allowing rapid construction of plasmids for the expression of gRNAs targeting any genomic locus of interest. Briefly, the whole sequence of pgRNA-bacteria was amplified using the designed forward and reverse primers carrying target gRNA sequence (Supplementary Table S1). After *Dpn*I digestion of template plasmid, the PCR fragment was phosphorylated by T4 polynucleotide kinase and ligated using DNA ligase. The sequence analysis of resultant plasmids (pgRNA-bacteria-*mreB*1 ~ 3) was performed to confirm gRNA insertion. The resultant plasmids and pdCas9-bacteria (Addgene ID:44251) were transformed into BW25113 WT and named *mreB*^R1 ~ 3^ strains (Table 1). BW25113 with pdCas9-bacteria and pgRNA-bacteria (empty plasmid) was also prepared as mock (*mreB*^C^).

**Table 1:** *Escherichia coli* strains and plasmids used in this study.

Strains and plasmids	Note	References
*E. coli*		
DH5α	F−, Φ80d *lacZ*ΔM15, Δ(*lacZYA*-*argF*)U169, *deoR*, *recA*1, *endA*1, *hsdR*17(rK− mK+), *phoA*, *supE*44, λ-, thi-1, *gyrA*96, *relA*1	General cloning uses
BW25113	*rrnB*T14 Δ*lacZ*WJ16 *hsdR*514 Δ*araBAD*AH33	Baba et al. (2006)
Δ*rodZ* (JW2500)	BW25113, Δ*rodZ*::*FRT-Km-FRT*	Baba et al. (2006)
Δ*rodZ*/p*rodZ*	Δ*rodZ*/pNTR-SD-*rodZ*	This study
*mreB*^C^	BW25113/pdCas9-bacteria/pgRNA-bacteria (mock)	This study
*mreB*^R1 ~ 3^	BW25113/pdCas9-bacteria/pgRNA-bacteria-*mreB*1 ~ 3	This study
**Plasmid**		
pNTR-SD-*rodZ*	pNTR-SD carrying *rodZ* (*yfgA*)	NBRP ID:2416
pgRNA-bacteria	Plasmid carrying cloning site for gRNA	Addgene ID:44249
pgRNA-bacteria-*mreB*1 ~ 3	pgRNA-bacteria carrying each gRNA for *mreB*	This study
pdCas9-bacteria	Plasmid carrying catalytically inactive Cas9	Addgene ID:44251

### Culture condition and gene expression analysis

The *E. coli* cells were cultured in lysogeny broth (LB; 10 g/L Bacto^™^ Tryptone, 5 g/L yeast extract, and 10 g/L NaCl). The culture medium for the strains harboring plasmids was supplemented with 50 mg/L chloramphenicol and/or ampicillin. All the test cultures were precultured in LB for 18 h at 37°C and inoculated into 100 mL of fresh LB in a 500 mL baffled conical flask to achieve the optical density at 660 nm (OD_660_) of 0.01. The cultures were incubated on an NR-30 rotary shaker (Taitec, Osaka, Japan) at 140 rpm. Cell growth was measured at OD_660_.

To examine gene expression, cells of each strain were harvested at log phase (7.5 h) post-inoculation by centrifugation at 4°C for 5 min at 8,000 × *g*. Total RNA was extracted from the collected cells as described elsewhere (Ojima et al., 2018), and then reverse-transcribed into cDNA using a PrimeScript RT reagent kit (Takara Bio Inc., Kusatsu, Japan). Gene expression was analyzed by qRT-PCR (CFX Connect, Bio-Rad Laboratories, Inc., CA, United States), as described in our previous study (Ojima et al., 2020). The expression level of target genes was normalized against that of *rrsA* encoding 16S rRNA (internal reference). The specific primer pairs used are listed in Supplementary Table S1.

### Isolation of OMV and evaluation of production

OMVs were isolated as previously described (Gujrati et al., 2014) with some modifications. After culture for 24 h, 100 mL of the *E*. *coli* culture was centrifuged at 10,000 × *g* for 10 min at 4°C to remove the cells. Then, the supernatant was passed through a 0.45-μm filter. Ammonium sulfate was added at the final concentration of 400 g/L to incubate for 1 h at room temperature to precipitate the contents. Crude OMVs were obtained via centrifugation at 11,000 × *g* for 30 min at 4°C. The crude extracts were dissolved in 1 mL PBS (pH 7.5) and concentrated using a CS100FNX ultracentrifuge (Hitachi Koki Co., Tokyo, Japan) at 109,000 × *g* for 1 h. The OMV pellets were resuspended in 100 μL of PBS. The resulting OMV samples were 1,000 times more concentrated than that in the original culture because of the volume decreasing from 100 mL to 100 μL. The OMV samples were placed onto a 400-mesh copper grid and negatively stained with 2% phosphotungstic acid (pH 7.0) for TEM observation under a JEM-1010 (JEOL, Tokyo, Japan).

To evaluate the OMV production, ten microliters of the isolated OMVs was analyzed via sodium dodecyl sulfate–polyacrylamide gel electrophoresis (SDS-PAGE) and Coomassie Blue staining. OMV production was quantified as previously described (Schwechheimer and Kuehn, 2013; Ojima et al., 2021) with some modifications. The OMV concentration was measured by quantifying the band at approximately 37 kDa in the SDS-PAGE gel using ImageJ software (National Institutes of Health, Bethesda, MD, United States). The OMV production of each mutant was shown as relative value to WT. OMVs were also quantified with a lipophilic dye FM4-64 according to a previously described method with minor modifications (Mcbroom et al., 2006; Manning and Kuehn, 2011; Klimentová and Stulík, 2015; Ojima et al., 2021). Isolated OMVs were incubated with 5 μg/mL FM4-64 (Molecular Probes/Thermo Fisher, Waltham, MA, United States) in PBS (pH 7.5) for 20 min. Then, OMVs were measured at the excitation and emission wavelengths of 558 and 734 nm, respectively, using an INFINITE 200 PRO spectrofluorophotometer (TECAN, Switzerland).

### Determination of OMV size using dynamic light scattering

OMVs isolated by ultracentrifugation as described above were resuspended in PBS (pH 7.5). After dilution of OMV samples with pure water, dynamic light scattering (DLS) measurements were conducted at 25°C using a ZetaSizer NanoSeries equipped with a HeNe laser source (λ = 633 nm) (Malvern Instruments Ltd., Worcestershire, UK) and analyzed using the Dispersion Technology Software (Malvern Instruments Ltd., UK). For each sample, the autocorrelation function was the average of five 10 s runs and then repeated approximately three to six times. CONTIN analysis was subsequently used for the number versus hydrodynamic size profiles to study the dispersions.

### Quick-freeze, deep-etch replica EM

*E. coli* cells were washed with PBS (pH 7.5) twice, resuspended in HEPES-NaCl buffer and centrifuged. QFDE-EM was performed as previously described (Ojima et al., 2021; Tahara and Miyata, 2023). Briefly, a rabbit lung slab, mica flakes, and *E. coli* cell pellets were placed in this order onto a paper disk attached to an aluminum disk, and the samples were quickly frozen using liquid helium with a CryoPress (Valiant Instruments, St. Louis, MO, United States). The rabbit lung slab and the mica flakes function as shock absorber in quick freezing and flat background in observation, respectively. The specimens were stored in a chamber at −180°C using a JFDV freeze-etching device (JEOL). After the samples’ temperature was increased to −120°C, they were freeze-fractured with a knife and freeze-etched at −104°C for 15 min. Subsequently, the samples were coated with platinum at a thickness of 2 nm and a rotary shadowing angle of 20° and then coated with carbon at a rotary shadowing angle of 80°. Next, the replicas were floated on full-strength hydrofluoric acid, rinsed in water, cleansed with commercial bleach containing sodium hypochlorite, rinsed in water, and finally placed onto 400-mesh Cu grids. The replica specimens were observed with transmission electron microscopy (TEM) using a JEM-1010 (JEOL).

### Cell volume distribution analysis using qNano system via scanning ion occlusion sensing

Cells of each strain were harvested at 24 h after inoculation by centrifugation at 10,000 × *g* and 4°C for 10 min. Cells were washed by filtered PBS (pH 7.5) and resuspended in PBS. Cell size distribution analysis was conducted using the qNano system (qNano, IZON Science Ltd., Christchurch, New Zealand) equipped with Nanopore NP-1000. Scanning ion occlusion sensing allows single-particle measurements since colloids or biomolecular analytes or both are driven through pores one at a time. Particles crossing the nanopore are detected as a transient change in the ionic current flow, also denoted as a blockade event whose amplitude is the blockade magnitude. As blockade magnitude is proportional to particle size, accurate particle sizing can be achieved after calibration with a known standard. Here size calibration was conducted using CPC1000 standard particles. It was guaranteed by the manufacturer that measurement is not affected by the optical properties or shape of the sample.

### Preparation of *Escherichia coli* PG and observation via QFDE-EM

After 24 h in culture, the *E*. *coli* cells were collected via centrifugation at 10,000 × g and 4°C for 10 min and suspended in PBS (pH 7.5). These steps were repeated once. Then, the cells were resuspended in 10% SDS (w/v) and incubated at 95°C for 12 h. The PG were harvested via centrifugation at 200,000 × *g* and 25°C for 40 min, washed twice in Milli-Q water, and observed using TEM with QFDE-EM.

### Culture with osmotic support

During the culture, 0.1–0.5 M sucrose was added to the LB medium when osmotic support was required for Δ*rodZ*. Cell growth and relative OMV production were evaluated after 24 h culture.

## Results

### Gene expression in CRISPRi-mediated strains

**Figure 1:**
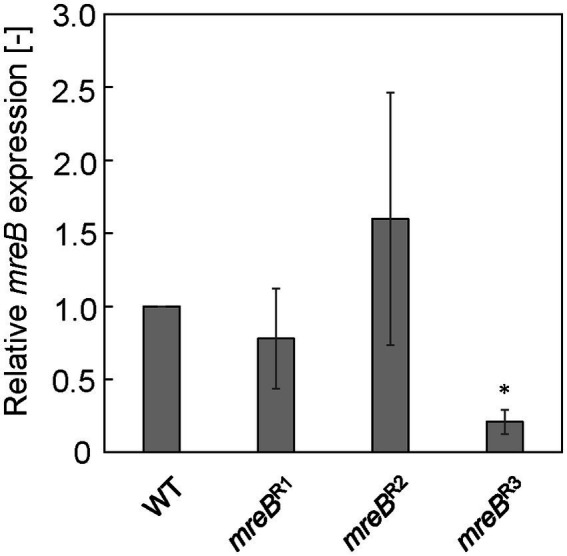
Relative *mreB* expression in *Escherichia coli* strains with CRISPRi system. The expression level was normalized against that in wild-type (WT) cells. The vertical bars indicate standard deviations (calculated from more than three independent experiments). Statistically significant difference from the WT strain (*p* < 0.05) is marked with asterisk.

Each *E. coli* strain was cultured in LB, and mRNA expression of *mreB* was evaluated by qRT-PCR at log phase (7.5 h). Figure 1 shows the relative expression level of *mreB* in each strain. The gRNAs constructed for repression of *mreB* generated different levels of repression. In the case of *mreB*^R1^ and *mreB*^R2^ cells, the *mreB* expression was not significantly repressed compared to WT cells. In addition, the variation of the value was very large. The *mreB*^R3^ showed the significant repression with small variation; the expression level of *mreB* was approximately 20% of that in WT cells, indicating that *mreB* expression was successfully repressed by using the CRISPRi system. From these results, *mreB*^R3^ was used in subsequent experiments.

### Cell growth, and OMV production and size distribution

**Figure 2:**
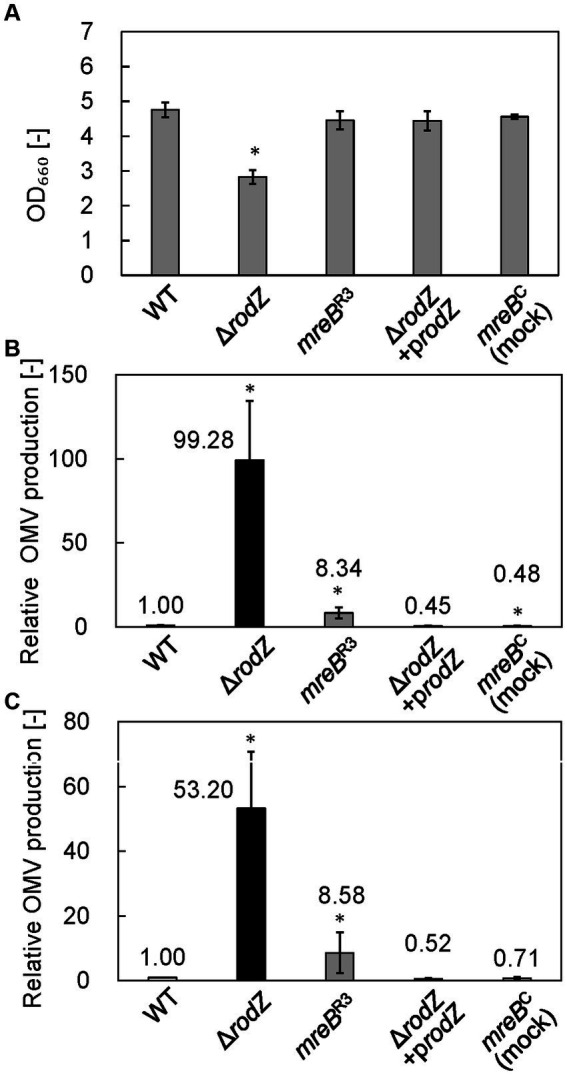
Cell growth and relative outer membrane vesicle (OMV) production by each *E. coli* strain. **(A)** OD_660_ values of culture of each *E. coli* strain after 24 h. **(B)** Relative OMV production by each *E. coli* strain, evaluated by staining with FM4-64 dye. **(C)** Relative OMV production by each *E. coli* strain, evaluated by sodium dodecyl sulfate–polyacrylamide gel electrophoresis (SDS-PAGE). OMV production was normalized to that by the WT strain. Vertical bars indicate standard deviations (calculated from more than three independent experiments). Statistically significant differences from the WT strain (*p* < 0.05) are marked with asterisks.

The cell growth and relative OMV production of each strain were analyzed after 24 h of culture in LB. Figure 2A shows the cell growth of each strain. Whereas the OD_660_ value of the WT strain was 4.76, that of the Δ*rodZ* strain was 2.83, which was significantly lower. The complemented strain Δ*rodZ*/p*rodZ* showed a restored OD_660_ value of 4.44. The OD_660_ values of strains *mreB*^C^ (mock repression of *mreB*) and *mreB*^R3^ were 4.56 and 4.46, respectively, suggesting that the plasmid introduction and repression of expression of *mreB* did not influence the cell growth.

OMV production was quantified by using a lipophilic dye, FM4-64, and by SDS-PAGE. The results analyzed by FM4-64 were summarized in Figure 2B. In the case of SDS-PAGE, the bands observed at ~37 kDa (OmpF, OmpC, and OmpA) provide an index of the amount of OMVs because these membrane proteins are expressed specifically and abundantly within the outer membrane of cells, and therefore predominate in OMVs (Schwechheimer and Kuehn, 2013). Supplementary Figure S2 shows the SDS-PAGE band of each OMV sample. Dilution rate of sample was changed depending on the OMV amount contained in each sample. Results clearly confirmed the presence of SDS-PAGE bands at ~37 kDa in samples from all strains, indicating that OMVs were successfully isolated from the culture broth. Next, we quantitatively analyzed OMV production on the basis of densitometry of the ~37 kDa SDS-PAGE bands (Figure 2C). The OMV production by the Δ*rodZ* strain assayed by using FM4-64 or SDS-PAGE was 99 and 53 times higher, respectively, than that by the WT strain (Figures 2B,C), consistent with a previous report (Kulp et al., 2015). Regarding OMV production, the relative value evaluated using FM4-64 dye was about twice that determined by SDS-PAGE, suggesting that the values differed depending on whether the OMV abundance was calculated based on lipids (using the dye) or outer membrane proteins (using SDS-PAGE).

Meanwhile, the OMV production by strain *mreB*^R3^ was determined to be approximately eight times that by the WT strain by both FM4-64 and SDS-PAGE analyses. This study suggests that repression of the expression of *mreB* increases OMV production by *E. coli*. In contrast, the complemented strain Δ*rodZ*/p*rodZ* and the mock-repression strain *mreB*^C^ did not show an increase in OMV production, supporting the finding that deletion of *rodZ* or repression of *mreB* expression enhanced the OMV production.

Next, isolated OMVs from each strain were observed by TEM with negative staining (Supplementary Figure S3A). OMV particles ~20–200 nm in diameter were observed for all strains, suggesting that OMVs were successfully isolated from culture broth. The diameter of the OMVs from each strain was measured via DLS, and the size distribution of the OMVs was plotted (Supplementary Figure S3B). The WT strain had a normal diameter distribution, with a peak value of approximately 8% at around 90 nm diameter; this distribution agreed with previous research (Alves et al., 2016). The average OMV size was 83.9 nm. The distribution of the sizes of the OMVs of the Δ*rodZ* and *mreB*^R3^ strains almost overlapped with that of the WT strain; the average sizes of the OMVs from the Δ*rodZ* and *mreB*^R3^ strains were 89.8 and 88.3 nm, respectively.

### Observation of *Escherichia coli* cells using QFDE-EM

**Figure 3:**
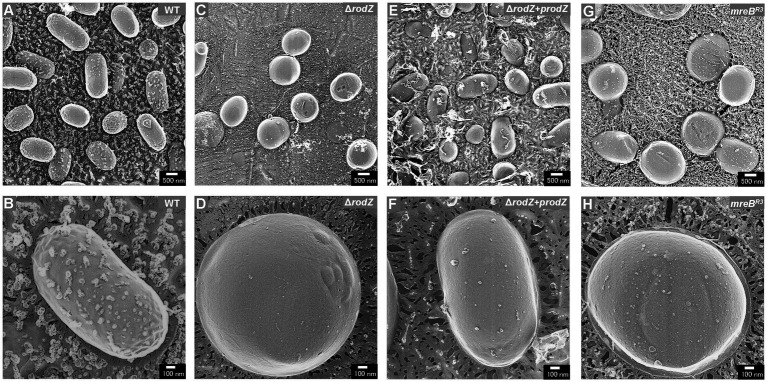
Surface structure of the *E. coli* WT, Δ*rodZ*, Δ*rodZ + prodZ* (complementary strain), and *mreB*^R3^ (expression of *mreB* repressed—see Figure 1) cells visualized via quick-freeze, deep-etch electron microscopy (QFDE-EM). Field image of WT **(A)**, Δ*rodZ* **(C)**, Δ*rodZ + prodZ* **(E)**, and *mreB*^R3^ **(G)** cells. Magnified image of the surface of WT **(B)**, Δ*rodZ* **(D)**, Δ*rodZ + prodZ* **(F)**, and *mreB*^R3^ **(H)** cells. The cells were collected after 24 h of culture.

QFDE-EM is a powerful tool for investigating the spatial structure of bacterial envelopment (Tulum et al., 2019); it has been applied to analyze the biogenesis of OMVs in *Buttiauxella agrestis* (Takaki et al., 2020) and *E. coli* (Aktar et al., 2021; Ojima et al., 2021). Here, the cellular structure (the surface of the cells) of each *E. coli* strain was visualized using QFDE-EM (Figure 3). In the case of WT cells, most of them were rod-shaped, although the cells varied in aspect ratio, which is normal for *E. coli* (Figures 3A,B). In contrast, the Δ*rodZ* cells appeared spherical, like a coccus (Figure 3C), which is consistent with the microscopic observation in a previous report (Bendezú et al., 2009). Magnified image demonstrated that the surface of the most Δ*rodZ* cells appeared larger and remarkably smooth (Figure 3D), suggesting that the cells were expanding by osmotic pressure. In the case of the complementary strain Δ*rodZ* + p*rodZ*, most cells returned to rod-shaped (Figure 3E). There were some cells appeared to be spherical. However, considering that they were also observed in WT cells with similar frequency, this is thought to be due to differences in the orientation of cells on the replica membrane. Magnified image showed the cell with normal aspect ratio (Figure 3F). As shown in Figure 3G, a spherical cell shape was also observed for strain *mreB*^R3^, as previously reported (Elhadi et al., 2016). Magnified images showed that strain *mreB*^R3^ cells were larger than WT cells (Figure 3H), indicating that the repression of *mreB* expression led to a similar phenotype to deletion of *rodZ*.

**Figure 4:**
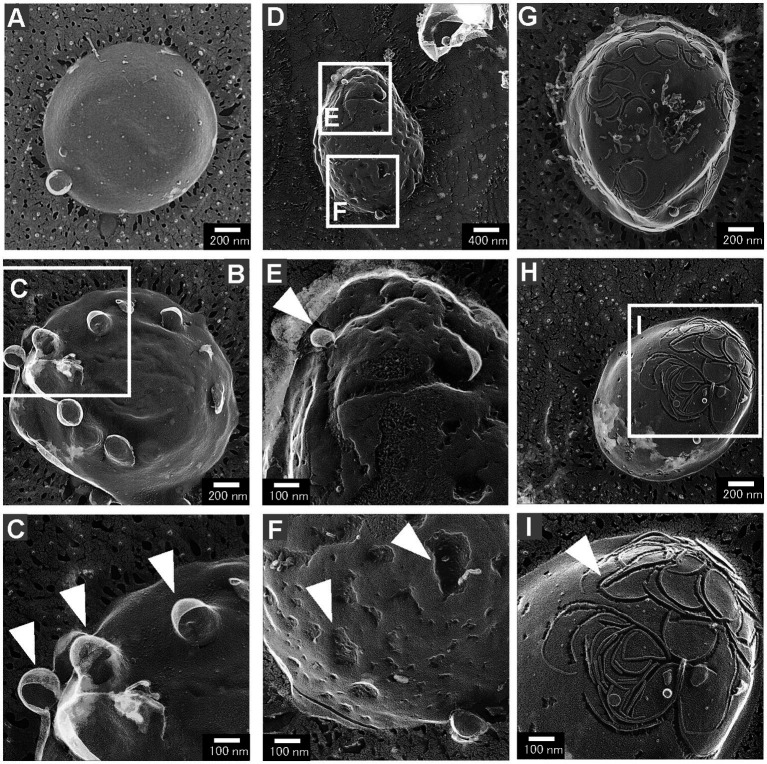
Aberrant surface structures of *E. coli* Δ*rodZ* cells. **(A,B)** Cells with a budding surface. **(C)** Magnified image of the surface structure of the budding vesicles. The arrowheads indicate vesicles budding from the cell surface. **(D)** Cells with the budding vesicles and a dented surface. **(E)** Magnified image of a budding vesicle; the arrowhead indicates the budding vesicle. **(F)** Magnified image of a dented surface; the arrowheads indicate the dents on the surface. **(G,H)** Cells with the curved pattern on the surface. **(I)** Magnified image of a curved pattern. The arrowhead indicates the curved pattern, which randomly appeared on the cell surface.

While most cells of the Δ*rodZ* strain had a smooth cell surface, some had aberrant cell surface structures (Figure 4). These aberrant cell surface structures could be roughly categorized into three groups: cells with budding vesicles; cells with a dented surface; and cells with a curved pattern on the surface. These types of cells were not observed for the *mreB*^R3^ strain. The cells with budding vesicles had vesicle-like structures budding from the cell surface (Figure 4A). Other cells had wrinkles and lots of budding vesicles on the cell surface (Figure 4B). Magnified images showed that the diameters of the vesicles were approximately 100–150 nm (Figure 4C), which corresponds to the size of OMVs (Supplementary Figure S3). The frequency of cells with budding vesicles was 3.7% (114 cells/3069 total cells). The cells with a dented surface had both budding vesicles and many hollows on the cell surface (Figure 4D), which has not been observed for WT cells or gene-deficient mutants studied in the past. Magnified images showed the budding vesicles and hollows on the cell surface both had diameters of approximately 100 nm (Figures 4E,F). These cells were observed at a frequency of 2.2% (68 cells/3,069 total cells). Cells with curved patterns on the surface are shown in Figures 4G,H. Many irregular curved patterns on the cell surface were observed in magnified images (Figure 4I). Although the proportion of these cells was low (1%, 30 cells/3,069 total cells), they have a very distinctive surface structure that has never been observed for *E. coli*. These three types of cells together accounted for approximately 7% of the total cells.

**Figure 5:**
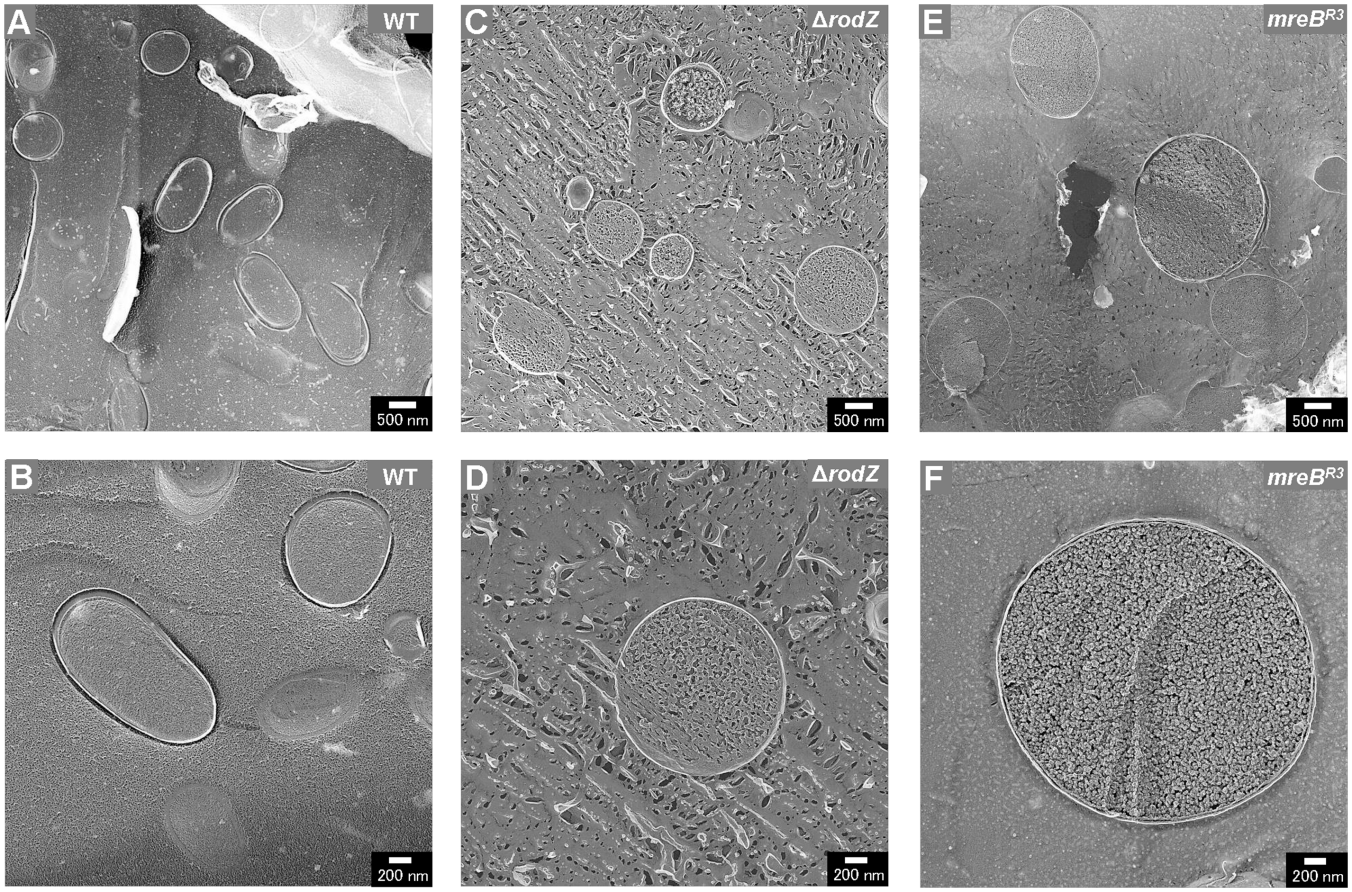
Cross-section of various fractured *E. coli* cells. Field image of the structure of WT **(A)**, Δ*rodZ* **(C)**, and *mreB*^R3^ **(E)** cells. Magnified image of the surface of WT **(B)**, Δ*rodZ* **(D)**, and *mreB*^R3^ **(F)** cells. The cells were collected after 24 h of culture.

Plasmolysis is the shrinking of protoplasm away from the cell wall of a bacterium and was reported to be key factor for OMV production by cells of other hypervesiculating *E. coli* strains such as Δ*nlpI*, Δ*mlaE* (Ojima et al., 2021). Next, the intracellular compartments of cells, i.e., the outer membrane, inner membrane, and cytoplasm, were visualized by cross-section observation using freeze-fractured sections without a freeze-etching step (Figure 5). The cross-section of WT cells was oval and, as previously reported, plasmolysis was not observed (Figures 5A,B). The cross-section of Δ*rodZ* cells was circular (Figure 5). Magnified images showed that most of the cells had a dense structure without a large periplasmic space (Figures 5C,D). The cross-section of the *mreB*^R3^ cells was similar to that of Δ*rodZ* cells (Figure 5E). The magnified images showed an extremely large cell with diameter of about 3 μm (Figure 5F).

### Cell volume of each *Escherichia coli* strain

**Figure 6:**
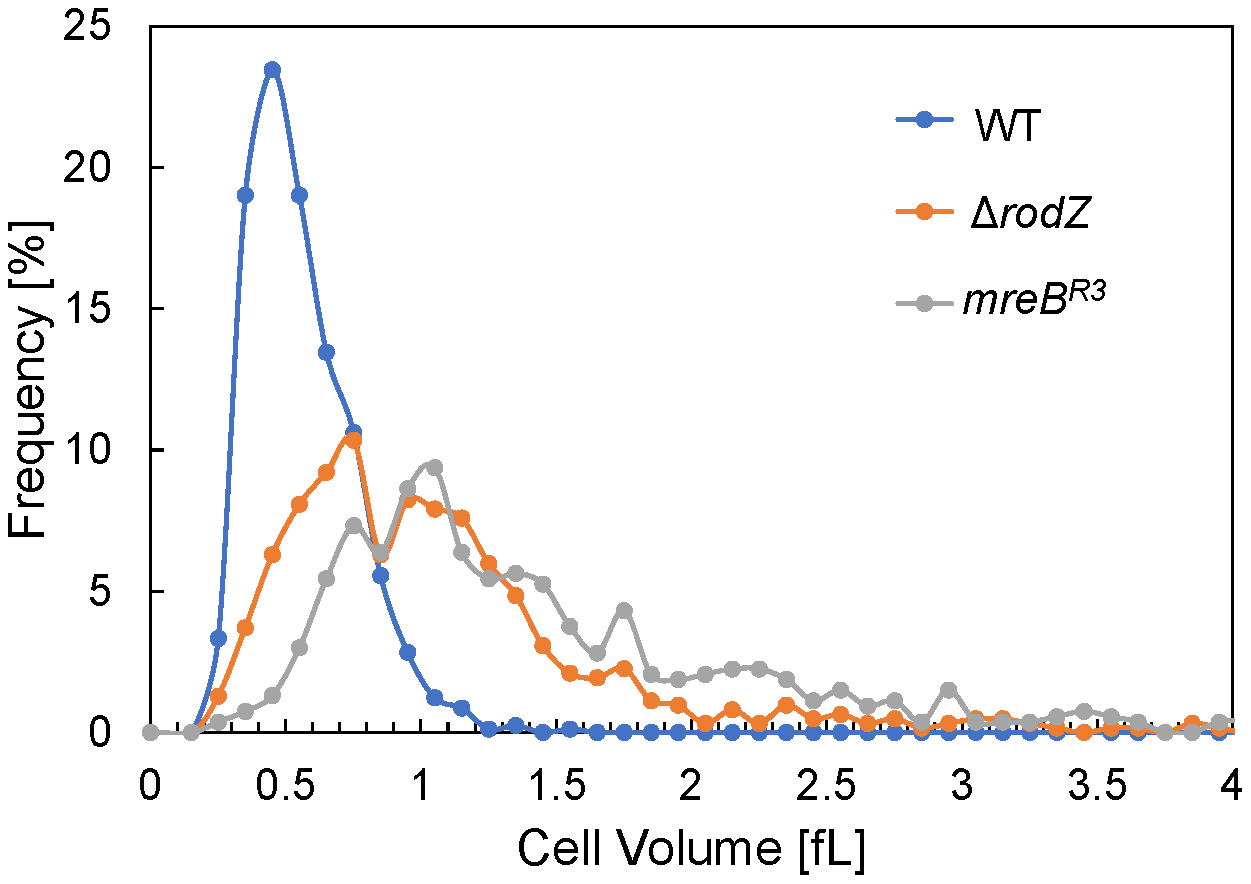
Distribution of cell volumes for each *E. coli* strain. The cells were harvested after 24 h of culture. Cell volume was determined using the qNano system.

QFDE-EM revealed that plasmolysis was not observed in Δ*rodZ* and *mreB*^R3^ cells, and these cells were expanding by the osmotic pressure. Therefore, the cell volume of each strain was measured after 24 h of culture using the qNano system and plotted in histograms (Figure 6). The cell volume of the WT strain was normally distributed in the range of ≤1 fL and the average volume was 0.56 fL, consistent with that in a previous study (Kubitschek, 1990; Ojima et al., 2021). In contrast, the plots for Δ*rodZ* and *mreB*^R3^ cells did not follow the normal distribution and the distribution shifted to a larger value than for the WT. Large cells, >3 fL, were detected for both strains. The average volume of cells of the Δ*rodZ* and *mreB*^R3^ strains was 1.13 and 1.45 fL, respectively. Thus, it was suggested that Δ*rodZ* and *mreB*^R3^ cells are not only spherical but also significantly expanded.

### Observation of PGs using QFDE-EM

Previous research has reported that the PG of Δ*rodZ* and *mreB*^R3^ *E. coli* mutants was circular and had holes that were observed by using QFDE-EM (Ago et al., 2023). Therefore, here, PG of each *E. coli* strain were prepared according to previous research (Vollmer et al., 2008; Jeske et al., 2015). The WT PG were observed using QFDE-EM (Supplementary Figure S4). The surface of the typical WT PG appeared oval and remarkably smooth (Supplementary Figure S4A). In contrast, the PG of the Δ*rodZ* strain were circular and had several holes on the surface (Supplementary Figure S4B). The diameter of the holes was >10 nm, substantially larger than the mean radius of the pores for *E. coli* PG in a previous report (2.06 nm) (Demchick and Koch, 1996). The holes in the PG were confirmed in many other cells. These holes were also observed for *mreB*^R3^ cells (Supplementary Figures S4C,D). These results suggest that the structure of the PG layer is vulnerable in both Δ*rodZ* and *mreB*^R3^ *E. coli* cells.

### Effect of osmotic support on cell growth and vesicle production by the Δ*rodZ* strain

**Figure 7:**
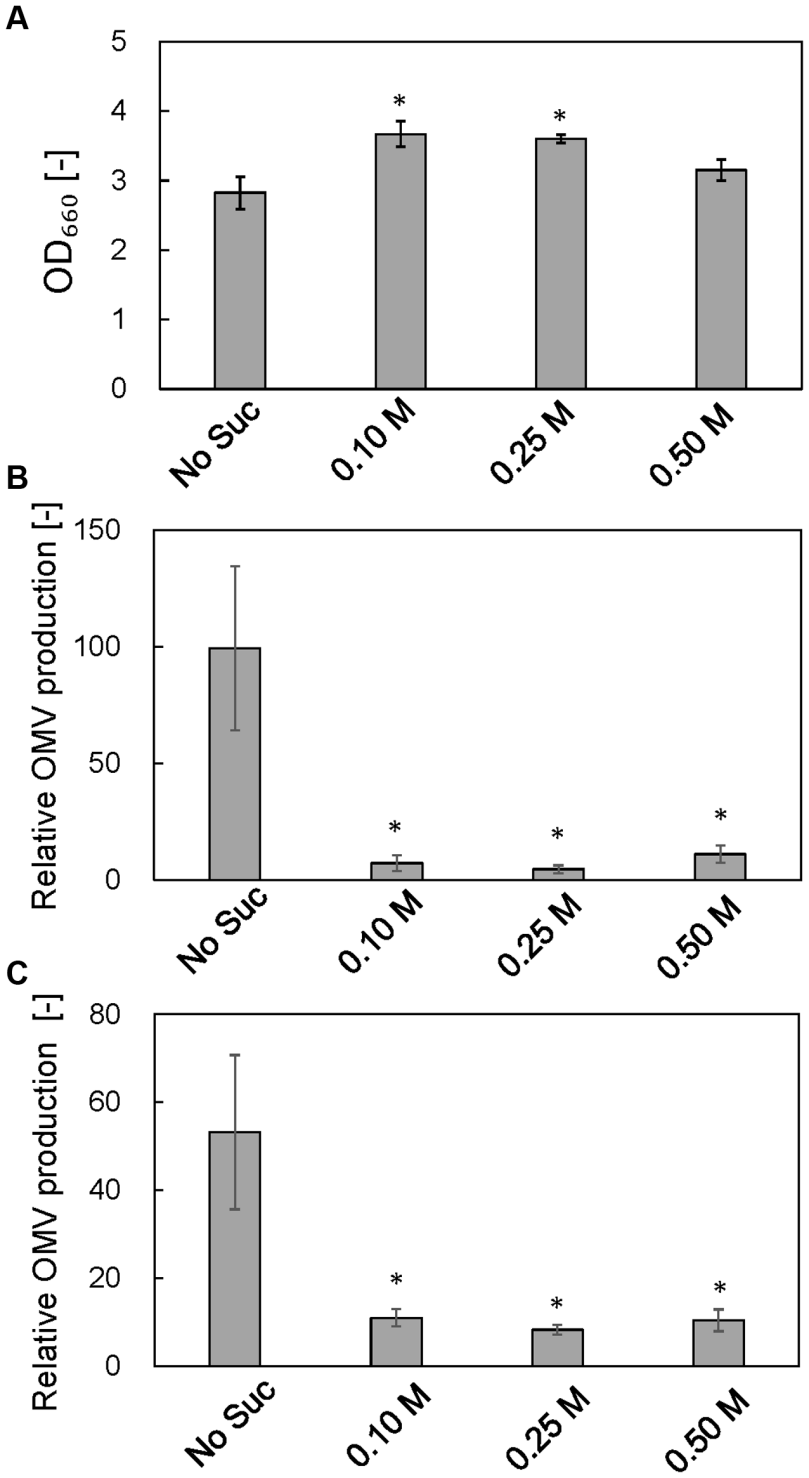
Effect of osmotic support on cell growth and relative OMV production by Δ*rodZ.* strain. **(A)** OD_660_ value of Δ*rodZ* strain after 24 h of culture with various concentrations of sucrose. **(B)** Relative OMV production by Δ*rodZ* strain evaluated by FM4-64 staining. **(C)** Relative OMV production by Δ*rodZ* strain evaluated by SDS-PAGE. OMV production was normalized to that by the WT strain without osmotic support. Vertical bars indicate standard deviations (calculated from more than three independent experiments). Statistically significant differences from No Suc (*p* < 0.05) are marked with asterisks.

To examine the osmotic effect on cell growth and vesicle production, Δ*rodZ* cells were cultured in LB with osmotic support—sucrose was supplemented to 0.1, 0.25, and 0.5 M (0.25 M sucrose is isotonic) (Figure 7). Whereas the OD_660_ value of Δ*rodZ* cell culture in LB was 2.83 after 24 h, the OD_660_ value was 3.67 and 3.60 in the presence of 0.1 and 0.25 M sucrose, respectively (Figure 7A). The OD_660_ value decreased again when the sucrose concentration was increased to 0.5 M. OMV production was evaluated by using both FM4-64 and SDS-PAGE. Using FM4-64, in LB, the relative OMV production by Δ*rodZ* cells was 99.28 times that by WT cells; that decreased to 7.17 times in the presence of 0.1 M sucrose, and to 4.57 times in the presence of 0.25 M sucrose, <1/20 of that without sucrose addition (Figure 7B). Further increasing the sucrose concentration to 0.5 M increased vesicle production again because these conditions are hypertonic. A similar trend was observed when the evaluation used SDS-PAGE (Figure 7C), and it was confirmed that osmotic support restored the growth of Δ*rodZ* cells and repressed vesicle production by this strain.

## Discussion

The properties of cells of the *E. coli* Δ*rodZ* and *mreB*^R3^ strains, and the OMVs that they produced, were compared with those of the WT, and a mechanism for the hypervesiculation of these strains was explored. First, repression of *mreB* expression was achieved using CRISPRi. We designed three gRNA sequences from the upstream side of the *mreB* sequence. Although it was reported that designing gRNA on the upstream side has higher repression efficiency (Fang et al., 2021), in our results, only *mreB*^R3^ with gRNA designed at the most downstream showed significant repression of *mreB* expression. These results suggest that the binding activity of gRNA to the target sequence on genome is the most important for stable repression of gene expression. In the culture of Δ*rodZ* and *mreB*^R3^ strains, while the OD_660_ value of culture of Δ*rodZ* cells was significantly lower than that of WT cells, the OD_660_ value of *mreB*^R3^ culture was comparable to that of the WT. Whereas OMV production by Δ*rodZ* cells was much higher than that by WT cells as previously reported, OMV production by *mreB*^R3^ cells was about eight times that by WT cells. This is the first report that repression of *mreB* expression increases OMV production.

Next, the cell structure of each strain was observed using QFDE-EM. Both Δ*rodZ* and *mreB*^R3^ cells were spherical, larger than WT cells. Some Δ*rodZ* cells had aberrant surface structures, such as budding vesicles, a dented surface. These results suggest that the budding vesicles are OMVs and the hollows on the cell surface are traces of budding. Cross-sections of the fractured cytosol and membrane surface showed that the spherical cells were not plasmolyzed. The Δ*rodZ* strain was shown to have a larger cell volume than the WT, suggesting that the spherical cells lacking an orientated PG structure were affected by osmotic pressure and expanded. *mreB*^R3^ cells were also larger than the WT, and even larger than Δ*rodZ* cells. Considering that the OD_660_ of culture of *mreB*^R3^ cells was significantly higher than that of Δ*rodZ* cells, *mreB*^R3^ cells can withstand further expanding without exploding. This is because both *rodZ* and *mreB* are expressed in the *mreB*^R3^ cells, although *mreB* is repressed by the CRISPRi. In addition, another group already reported that the shape of PG isolated from Δ*rodZ* cells was circular with many holes (Ago et al., 2023), suggesting that *rodZ* may be a determinant not only of the whole shape of the PG layer but also of its very dense structure.

From these results, we hypothesize that OMV production by the Δ*rodZ* strain was enhanced by both a budding cell surface and cell burst due to osmotic pressure. Our hypothesis was verified by culturing the Δ*rodZ* strain with osmotic support. Sucrose was selected to provide osmotic support because *E. coli* cannot assimilate sucrose (Lee et al., 2010). In conditions of osmotic support, the OD_660_ value of the Δ*rodZ* strain was significantly restored, and OMV production drastically decreased, indicating that osmotic support repressed the budding on the cell surface and the cell burst. However, the OD_660_ value for the Δ*rodZ* strain was still lower than that of the WT even in conditions of osmotic support. Because the Δ*rodZ* strain has been reported to have a decreased ability to use carbon sources (Ito et al., 2005), it seems that osmotic support was unable to completely restore its growth.

**Figure 8:**
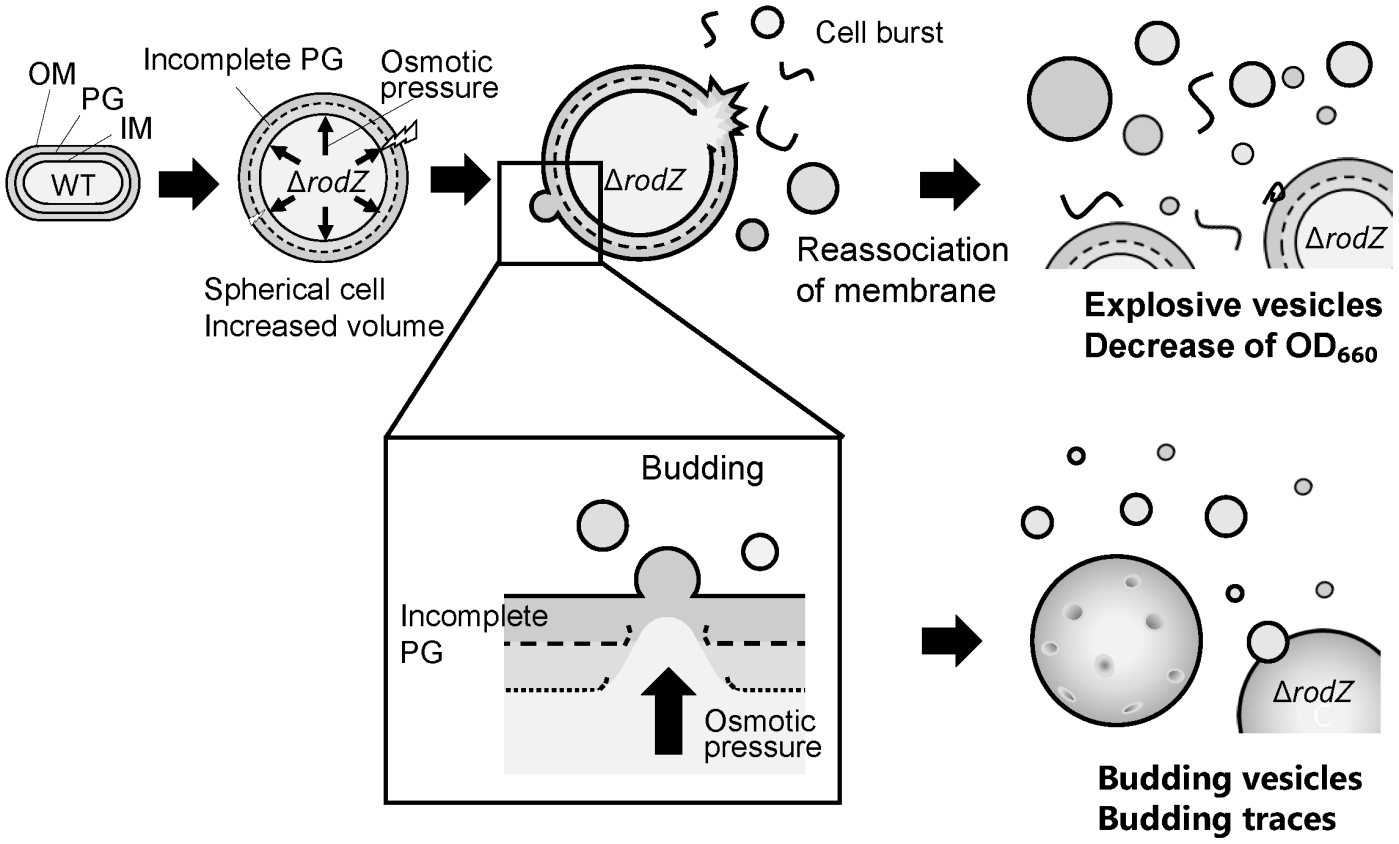
Model of the proposed mechanism underlying the enhanced production of vesicles by the *E. coli* Δ*rodZ* strain. The Δ*rodZ* cells become spherical and expanded by osmotic pressure due to their incomplete peptidoglycan (PG) structure; budding vesicles are generated from the sites of holes in the PG. Budding traces were also observed by QFDE-EM. In parallel, cell burst and the reassociation of membrane fragments result in explosive vesicle production. This model was supported by the restored OD_660_ value and decreased vesicle production in conditions of osmotic support.

On the basis of these findings, a model of the hypervesiculation mechanism of the Δ*rodZ* strain is proposed (Figure 8). In this model, the Δ*rodZ* cells become spherical because of the loss of orientation of PG and expand by osmotic pressure. Budding vesicles on the surface of Δ*rodZ* cells and holes in the PG of Δ*rodZ* cells were observed. It seems that budding of vesicles occurs from the sites of these PG holes on the spherically expanded Δ*rodZ* cells. Regarding the influence of osmotic pressure on budding, it was reported that increasing the osmotic pressure delayed the budding time of yeast cells by decreasing the cell volume (Nishino et al., 1985). It can be concluded that, here, osmotic support inhibited the budding of *E. coli* Δ*rodZ* cells, resulting in decreased vesicle production. In the case of explosive vesicles, some expanded cells burst because of osmotic pressure; the shattered membrane fragments self-assemble into MVs. In research on *Pseudomonas aeruginosa*, cell burst was initiated by the activity of a cryptic prophage endolysin, acting as a mechanism for explosive OMV production (Turnbull et al., 2016). In this study, as a different mechanism from cell lysis by prophage, we revealed that *E. coli* cells with incomplete PG produce explosive vesicles by osmotic pressure.

Thus, this study first reported the mechanism that the vesicle production in *E. coli* Δ*rodZ* cells is promoted by surface budding and burst of cells that have become osmotically sensitive because of the incomplete PG structure.

## Data availability statement

The original contributions presented in the study are included in the article/Supplementary material, further inquiries can be directed to the corresponding author.

## Author contributions

YO: Writing – review & editing, Writing – original draft, Visualization, Supervision, Resources, Data curation, Conceptualization, Funding acquisition. KT: Writing – review & editing, Visualization, Methodology, Investigation, Formal analysis. TS: Writing – review & editing, Resources, Methodology. YK: Writing – review & editing, Resources, Methodology. YT: Writing – review & editing, Visualization, Software, Methodology, Formal analysis. MM: Writing – review & editing, Validation, Supervision, Methodology, Funding acquisition. MA: Writing – review & editing, Validation, Supervision, Methodology.
