# Supplementary material for: Budding and explosive membrane vesicle production by hypervesiculating Escherichia coli strain ΔrodZ
Source: Front Microbiol. 2024 Jun 20;15:1400434. doi: 10.3389/fmicb.2024.1400434 (PMC11222570; doi:10.3389/fmicb.2024.1400434)
Supplement: Supplementary file 1 [file Data_Sheet_1.PDF]

Supplementary Table S1 Primers used in this study

Primers	Sequences
Inverse PCR for gRNA	
mreB -F1	5′- GCGCTCACAATACCGGTCAGGTTTTAGAGCTAGAAATAGCAAGTTAAAATAAGGC -3′
mreB -F2	5′- GGAGATAACAGCAACTTCAGGTTTTAGAGCTAGAAATAGCAAGTTAAAATAAGGC -3′
mreB -F3	5′- ATCCTGACGAATGGCCACCAGTTTTAGAGCTAGAAATAGCAAGTTAAAATAAGGC -3′
mreB -R	5′- ACTAGTATTATACCTAGGACTGAGCTAGC -3′
qRT-pPCR	
rrsA-F (internal reference)	5′- TACGACCAGGGCTACACACG -3′
rrsA-R (internal reference)	5′- ATCCGGACTACGACGCACTT -3′
mreB-F	5′- CAATGCGCACAGAAGAGGAG -3′
mreB-R	5′- CGACCGGTTCTATGGTGGTT -3′

Supplementary Fig. S1 Design of target sequences for *mreB* in CRISPRi.

mreB

5′-

TTACTCTTCGCTGAACAGGTCGCCGCCGTGCATGTCGATCATTTCCAGCGCTTTGCCGCCACCGCGCGCC

ACACAGGTCAGCGGGTCTTCAGCAACAACGACTGGAATGCCGGTTTCTTCCATTAACAAACGGTCAAGGT

TACGCAGCAGTGCGCCACCACCGGTGAGCACCATGCCGCGCTCGGAGATGTCGGAAGCCAGTTCCGGCGG

GCACTGTTCCAGTGCAACCATTACCGCGCTCACAATACCGGTCAGCGGTTCCTGCAGTGCTTCGAGGATT

TCATTGGAGTTCAGGGTAAAACCGCGTGGAACACCTTCTGCCAGGTTACGGCCACGAACTTCGATTTCAC

GGACTTCATCGCCCGGATAAGCCGAACCGATTTCGTGCTTGATACGTTCTGCGGTGGCTTCACCGATCAG

AGAACCGTAATTACGACGCACATAGTTGATGATAGCTTCGTCGAAACGGTCACCACCAATGCGCACAGAA

GAGGAGTAAACCACACCGTTCAAGGAGATAACAGCAACTTCAGTGGTACCACCACCGATATCAACCACCA

TAGAACCGGTCGCTTCAGAAACCGGCAGGCCAGCACCAATTGCGGCAGCCATCGGTTCTTCAATCAGGAA

GACTTCACGGGCACCAGCGCCCTGCGCGGATTCACGAATTGCGCGGCGTTCAACCTGGGTCGCGCCAACC

GGCACACAAACCAGAACGCGCGGGCTTGGACGCATAAAGCTGTTGCTGTGCACTTGTTTGATGAAGTGCT

GGAGCATTTTTTCAGTCACGAAGAAGTCGGCGATAACGCCGTCTTTCATTGGGCGAATGGCAGCAATATT

GCCCGGCGTACGGCCCAGCATCTGCTTCGCGTCATGACCTACTGCAGCTACGCTTTTCGGTGAACCGGCA

CGATCCTGACGAATGGCCACCACGGAAGGCTCATTCAATACGATGCCTTGTCCTTTTACATAAATGAGGG

TATTCGCAGTACCCAGGTCAATGGACAAGTCATTGGAAAACATGCCACGAAATTTTTTCAACAT

-3′

<u>Inverse PCR for gRNA Primer</u>

Rank2:GCGCTCACAATACCGGTCAGCGG

→ *mreB*-F1 GC:42% Tm:80.9℃

GCGCTCACAATACCGGTCAGGTTTTAGAGCTAGAAATAGCAAGTTAAAATAAGGC

Rank10:GGAGATAACAGCAACTTCAGTGG

→ *mreB*-F2 GC:36% Tm:77.1℃

GGAGATAACAGCAACTTCAGGTTTTAGAGCTAGAAATAGCAAGTTAAAATAAGGC

Rank11:ATCCTGACGAATGGCCACCACGG

→ *mreB*-F3 GC:40% Tm:80.8℃

ATCCTGACGAATGGCCACCAGTTTTAGAGCTAGAAATAGCAAGTTAAAATAAGGC

<u>Real Time PCR Primer</u>

mreB-F

：CAATGCGCACAGAAGAGGAG GC:55% Tm:66.0℃

mreB-R

：CGACCGGTTCTATGGTGGTT GC:55% Tm:65.8℃

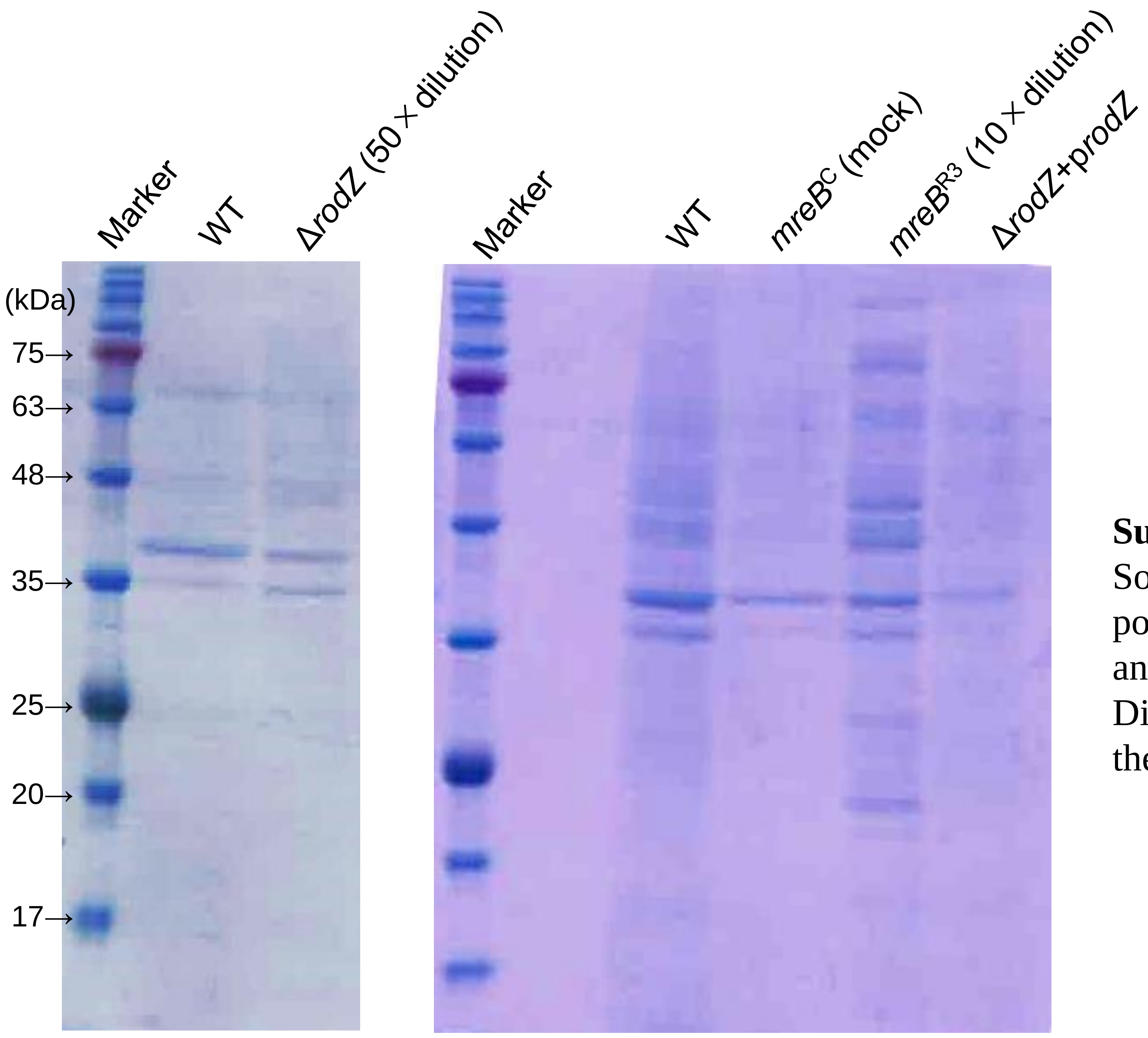

Supplementary Fig. S2
Sodium dodecyl sulfate-polyacrylamide gel electrophoresis (SDS-PAGE) analysis of OMVs isolated from each *E. coli* strain. Dilution rate of sample was changed depending on the OMV amount contained in each sample.

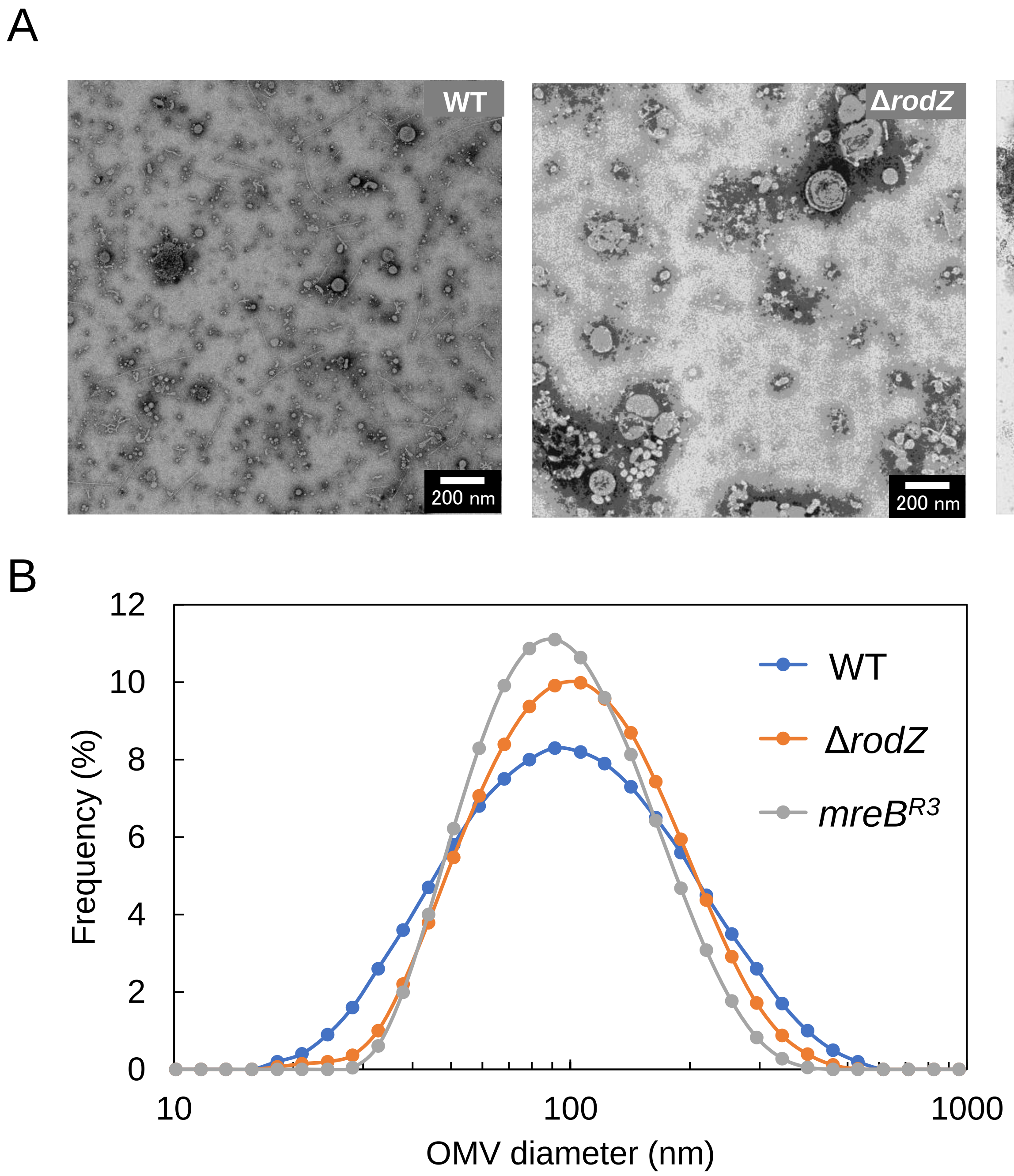

Supplementary Fig. S3

(A) Transmission electron microscopy images of the OMVs isolated from each *E. coli* strain. The OMVs were stained with 2% phosphotungstic acid (pH 7.0).

(B) Distribution of OMV diameter for each *E. coli* strain. The OMV samples were collected after 24 h of culture. The diameter of the OMVs was determined by using dynamic light scattering.

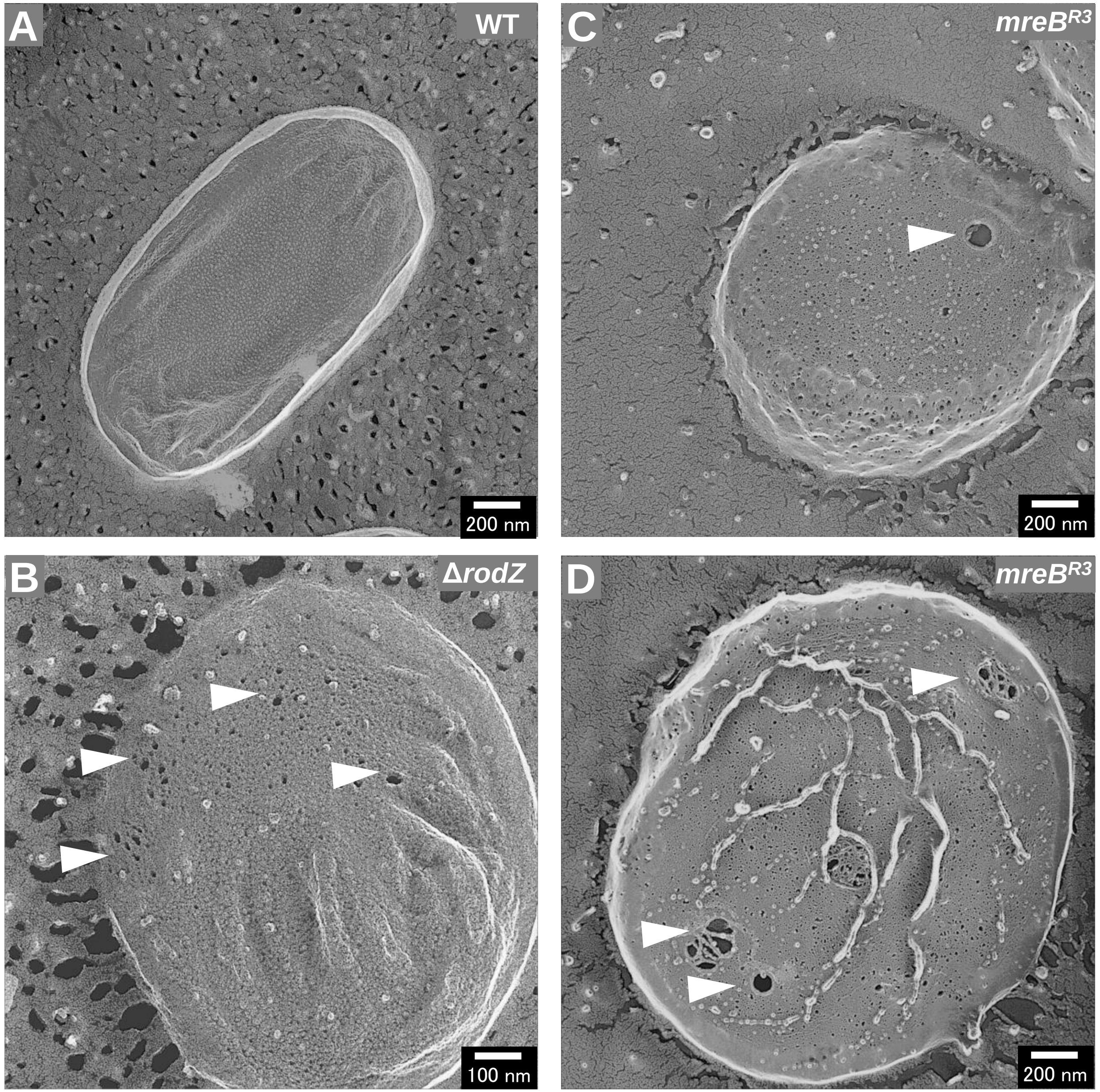

Supplementary Fig. S4

PG of each *E. coli* cell. The surface structures of the PG were visualized by QFDE-EM.
